# Bipolar disorder, cardiac comorbidity and therapeutic impasse: A case report

**DOI:** 10.1192/j.eurpsy.2021.1645

**Published:** 2021-08-13

**Authors:** I. Bouslama, H. Jemli, A. Ben Cheikh Ahmed, F. Nacef, R. Jomli

**Affiliations:** 1 Avicenne, manouba psychiatry, manouba, Tunisia; 2 Faculty Of Medicine Of Tunis, university of tunis elmanar, tunis, Tunisia

**Keywords:** bipolar disorder, comorbidity, cardiac disease

## Abstract

**Introduction:**

BipolarDisorders (BD) are regarded as a multidimensionaldiseaseinvolvingbothpsychological and physicaldeterminants. Althoughmood dimension andthymicinstability areconsidered as the « core » aspect of bipolardisorders, itis crucial to note thatsomaticproblemsfrequentlyoccur in BD,deeplyworsening the prognosis.

**Objectives:**

Herewedescribea case of atwentyyearshistory of psychiatricimpairment, diagnosedlaterwithcardiac malformation.

**Methods:**

Female patient H.G has been admitted for the first time to psychiatric department ‘A’ of Razi Hospital,treated for type 1 bipolar disorder since 2004 with poor therapeutic compliance. We reviewed the clinical and paraclinical data.

**Results:**

The patient was hospitalized for a severe manic episode with psychotic features, without cardiac personal history. The patient was asymptomatic and physical examination showed no abnormalities. Following a routine electrocardiogram, an acute coronary syndrom was discovered (inverted T waves seen in V1 to V6). Cardiac troponins were not elevated. According to cardiology recommandations, ischemic heart disease could not be ruled out and extensive cardiovascular investigations were needed. Antipsychotics and mood stabilizors were contraindicated.Therefore, the manic episode could only be managed using benzodiazepines. Given contradictions between clinical, electrocardioagraphic and imaging findings,coronary angiography was necessary. Results showed no significant stenosis of coronary arteries and a myocardial bridging of the left anterior descending artery and we were able to put her on antipsychiotics and moodstabilizer, almost two months after her admission.

**Conclusions:**

This case underlines the significant impact of somatic comorbidities in therapeutic management of bipolar disorders. Cardiovascular diseases in particular cause a delay in treatment initiation and an increase in patient length of hospital stay.

**Disclosure:**

No significant relationships.

